# Utilization patterns of malaria chemoprophylaxis among Tanzanian children attending sickle cell clinic in Dar es Salaam tertiary hospitals

**DOI:** 10.1186/s12936-019-3029-y

**Published:** 2019-12-03

**Authors:** Esther J. Ndegeulaya, George M. Bwire, Raphael Z. Sangeda, Doreen Mloka, Faustine Tungaraza, Augustino S. Kahere, Fidelis F. Manyaki, Fatuma F. Felician, Manase Kilonzi, Wigilya P. Mikomangwa, Hamu J. Mlyuka, Alphonce I. Marealle, Ritah Mutagonda, Liberata Mwita, Kennedy D. Mwambete

**Affiliations:** 1 Department of Pharmaceutical Microbiology, School of Pharmacy, Muhimbili University of Health and Allied Sciences, P.O. Box 65013, Dar es Salaam, Tanzania; 2 Department of Clinical Pharmacy and Pharmacology, School of Pharmacy, Muhimbili University of Health and Allied Sciences, P.O. Box 65013, Dar es Salaam, Tanzania

**Keywords:** Malaria, Chemoprophylaxis, Sickle cell, Children, Dar es Salaam

## Abstract

**Background:**

Malaria is among the leading cause of infection in individuals with sickle cell disease (SCD) living in sub-Saharan Africa, including Tanzania. However, after 2005 the standard treatment guidelines (STGs) on malaria chemoprevention for SCD patients were non-existent, and at present no medicine is recommended for SCD patients. Since several anti-malarials have been approved for the treatment of malaria in Tanzania, it is important to establish if there is a continued use of chemoprevention against malaria among SCD children.

**Methods:**

A cross-sectional, hospital-based study was conducted between January and June 2019 at tertiary hospitals in Dar es Salaam. Data were collected using a semi-questionnaire and analysed using SPSS software version 25. The descriptive statistics were summarized using proportions, while factors associated with the use of chemoprophylaxis were analysed using multivariate logistic regression. Statistical significance of p < 0.05 was accepted.

**Results:**

A total of 270 SCD children were involved. The median age of SCD children was 6 years (interquartile range (IQR): 3–11 years). Of 270 SCD children, 77% (number (n) = 218) of children with SCD had not been diagnosed with malaria in the previous year, whereas 12.6% (n = 34) of children were admitted because of malaria in the previous year. Regarding the use of chemoprophylaxis in SCD children, 32.6% (n = 88) of parents were aware that, chemoprophylaxis against malaria is recommended in SCD children. Of the 270 participants, 17% (n = 46) were using malaria chemoprophylaxis. A majority used artemisinin combination therapy (ACT), 56.8% (n = 26). Of 223 parents who did not give chemoprophylaxis, the majority (n = 142, 63.7%) indicated unavailability at clinics as the reason. Children whose parents were primary level educated were 9.9 times more likely to not use chemoprophylaxis (adjusted odds ratio (AOR); 9.9, 95% CI 1.8–56.5, P = 0.01) compared to those whose parents had tertiary education.

**Conclusion:**

Despite the lack of STGs, a small proportion of children with SCD were using malaria chemoprophylaxis where the majority used ACT, i.e., dihydroartemisinin-piperaquine.

## Background

Pregnant women and children, under 5 years old [1], including children with sickle cell disease (SCD) [2], are the groups mostly affected with malaria. The highest prevalence of SCD is found in tropical regions, particularly sub-Saharan Africa, India and the Middle East [3].

About 75% of 300,000 births of affected children globally live in sub-Saharan Africa; Tanzania holds fifth position in Africa with a high prevalence of children born with SCD [4]. Of the reported infections in a SCD population, malaria is among the leading causes of death in those who die before adulthood [5].

Different African studies suggest chemoprophylaxis against malaria is one of the approaches that may prevent malaria infection in SCD [6–9]. Furthermore, use of malaria chemoprophylaxis has been reported to reduce the number of crises, hospitalizations and episodes of anaemia [9].

In Tanzania malaria is endemic [10]; 93% of the population in Tanzania Mainland and the entire population of Zanzibar live in areas where malaria is transmitted [11]. Since 2005 there have been no standard treatment guidelines (STGs) (2007 through 2017), and at present no medicine is recommended for SCD as chemoprophylaxis. Furthermore, no alternative approaches have been suggested [12]. This study was conducted to assess the ongoing utilization of chemoprophylaxis against malaria in children with SCD.

## Methods

### Study design, area and population

A hospital-based, cross-sectional study was conducted between January and June 2019 in Dar es Salaam tertiary hospitals, including Muhimbili National Hospital (MNH), Mwananymala Referral Hospital, Amana and Mloganzila Hospital, to determine the utilization pattern of chemoprophylaxis against malaria in children with SCD. All children with SCD below 18 years of age attending sickle cell clinic in Dar es Salaam tertiary public hospitals were eligible to participate in this study. Tertiary hospitals are the only hospitals in Dar es Salaam to conduct weekly sickle cell clinics.

### Sample size and sampling technique

The sample size was calculated using single population proportion formula considering 95% confidence interval (CI) and proportion (P) of 0.6% [5] with 0.9% margin of error as follows;

$$n = {\text{Z}}_{{\upalpha /2}} {\text{P}}(1 - {\text{P}})/\upvarepsilon ^{2}$$

where n is sample size, Z_α/2_P = 1.96 for 95% confidence level, ε is the marginal error. Some 270 children were enrolled at the ratio of 1:1:1:1 (~ 68 children) from each clinic. The random sampling technique was employed to select the study participants. For each SCD clinic, the total number of registered SCD children was used to calculate the sampling interval “n” by taking the total number of registered SCD children at the respective clinic divided by 68. Participants were systematically selected after every “n” interval.

### Questionnaire development and validation

The semi-structured questionnaire consisted of 13 questions covering three major areas: (i) general information and consent/assent; (ii) social demographics of participants (parents and children); and, (iii) utilization of chemoprophylaxis against malaria in SCD population. The questionnaires were developed after a comprehensive literature review of the studies highlighting the need for malaria chemoprophylaxis against malaria in SCD children [6–9]. The questionnaires were written in English and translated in Swahili, the national language of Tanzania for convenient and accurate data collection. Ten individuals with firm command of both languages checked the accuracy and meaning of the translated content. The questionnaires were first validated and tested for answering the objectives of the study by randomly selected physicians and parents of SCD children in a pilot study (10 physicians, 10 parents). In scoring the frequency of malaria infection per year per child: no malaria, 1–3 times, more than three times were assigned as less common, common and more common, respectively.

### Data management and analysis

All the questionnaires were manually checked and incomplete questionnaires were not included in the final analysis. All the completed questionnaires were entered into Microsoft Excel and the data were exported to Social Statistical Package for Social Sciences (SPSS software version 25, Chicago Inc, USA) for analysis. The descriptive statistics were summarized using median, frequency distribution tables and proportions. Factors associated with the use of chemoprophylaxis as the primary outcome were measured by odds ratios using multivariate logistic regression. The result was considered of having statistical significance when P < 0.05.

### Ethical consideration

The ethical clearance to conduct this study was sought from MUHAS Research and Publication Committee (Ref No. DA.25/111/01). The permission to access participants from sickle cell clinics to fill the questionnaires was requested from the respective hospital authorities. The aim of the study was explained to parent/legal guardians before obtaining their written consent. Verbal assent was obtained from children above 5 years old before requesting consent from their parents/legal guardians. Participants were assigned a unique code and no names were recorded in the collected data for privacy.

## Results

### Participants’ social demographic information

A total of 270 SCD children were involved in this survey. The median age of SCD children was 6 years [interquartile range (IQR); 3–11]; children below age 5 years were more prevalent (44.1%). Children whose parents had a primary education were in the majority (48.1) and most of the parents (69.4%) were not employed (Table 1).

**Table 1:** Participant information

Characteristics	Frequency	Percentage (%)
Child’s age (years)		
≤ 5	119	44.1
6–12	104	38.5
13–18	47	17.4
Child’s gender		
Male	135	50
Female	135	50
Parent’s education		
Informal education	10	3.7
Primary education	130	48.1
Secondary education	106	39.3
Tertiary education	24	8.9
Parent’s occupation		
Employed	31	11.6
Self employed	51	19.0
Non employed	68.9	69.4

### Awareness of the use of chemoprophylaxis against malaria

Most of the parents (n = 234, 86.7%) of SCD children who participated in this survey reported that malaria was not a common infection in their children. On the other hand, 12.6% (n = 34) of SCD children were reported to have been admitted because of malaria in the previous year. Regarding the use of chemoprophylaxis, 32.6% (n = 88) of parents knew that their children were supposed to be given chemoprophylaxis and 17% (n = 46) of parents give their children chemoprophylaxis against malaria (Table 2).

**Table 2:** Malaria in sickle cell diseased children

Question	Frequency (n)	Percentage (%)
Is malaria a common infection to your child?		
Yes	36	13.3
No	234	86.7
How often does you child get malaria annually?		
Less common	208	77.0
Common	43	15.9
More common	19	7.0
Has your child been admitted because of malaria infection in the past year?		
Yes	34	12.6
No	236	87.4
Do you know that your child is supposed to use chemoprophylaxis against malaria?		
Yes	88	32.6
No	182	67.4
Does your child use malaria chemoprophylaxis?		
Yes	46	17.0
No	223	83.0

### Utilization pattern of chemoprophylaxis against malaria

Of 270 participants, 17% (n = 46) reported the use of chemoprophylaxis against malaria. The majority, 56.8% (n = 26), reported the use of artemisinin combination therapy (ACT). Surprisingly, 3 children reported the use of chloroquine (CQ) as chemoprophylaxis against malaria while 36% of children were using sulfadoxine-pyrimethamine (SP) although all of them are not in current guidelines for the treatment of malaria. Being not provided at the clinic was the reason reported by the majority (n = 142, 63.7%) of parents who did not provide chemoprophylaxis to their children (n = 223, 83%) (Table 3).

**Table 3:** Chemoprophylaxis utilization patterns against malaria

Question	Total number N = 270		Frequency	Percentage (%)
Does your child use malaria chemoprophylaxis?	Yes (n = 46)	Drug used		
		CQ	3	7.2
		SP	17	36.0
		ACT	26	56.8
	No (n = 223)	Reason		
		I was not aware	30	13.3
		Not provided at clinic	142	63.7
		Malaria is not a problem to my child	51	23.0

### Multivariate analysis

The dependent variable was, “does your child use malaria chemoprophylaxis? (Yes/No)”. Children whose parents had a primary education were more likely not using chemoprophylaxis (AOR 9.9, 95% CI 1.8–56.5, P = 0.01) compared to children whose parents had the tertiary level of education. Other factors, such as child’s age and parents’ occupation did not significantly influence the use of chemoprophylaxis against malaria (P > 0.05) (Table 4).

**Table 4:** Multiple-logistic regression analysis of factors associated with the use malaria chemoprophylaxis

Factor	Multivariate analysis AOR (95%CI), P value
Child’s age (years)	
Median (IQR); 6 (3–11)	0.8 (0.4–1.5), 0.465
Parent’s education	
Informal education	–
Primary education	9.9 (1.8–56.5), 0.01*
Secondary education	2.8 (0.6–13), 0.184
Tertiary education	–
Parent’s occupation	
Employed	2.1 (0.5–9.6), 0.345
Self employed	–
Unemployed	1.1 (0.34–3.3), 0.916
Malaria is a common infection in my child	
Yes	0.3 (0.0–1.42), 0.121
No	–
Malaria frequency	
Less common	0.7 (0.2–2.7), 0.639
Common	–
More common	0.1 (0.0–1.8), 0.714
Has your child been hospitalized due to malaria this year?	
Yes	–
No	1.1 (0.3–4.6), 0.869
Are you aware that your child needs malaria chemoprophylaxis?	
Yes	–
No	0.3 (0.0–5.8), 0.446

–, not estimable

* Statistically significant

## Discussion

This study was conducted to assess the current situation on chemoprevention against malaria in SCD children, following the lack in Tanzania of standard treatment guidelines [12].

A majority of the participants reported that their children were not commonly infected with malaria; this may be due to low prevalence (1.1%) reported in Dar es Salaam [11]. Furthermore, a small proportion of participants was both aware and were using chemoprophylaxis against malaria, contrary with the current policy which indicates no use of chemoprophylaxis against malaria in children with SCD [12]. Moreover, the study found the use of three anti-malarials: ACT, SP and CQ; the majority of participants were using ACT followed by SP. This contradicted the current treatment guidelines where ACT, especially artemether plus lumefantrine and dihydroartemisinin plus piperaquine, is recommended for the treatment of uncomplicated malaria in Tanzania, while SP is reserved for intermittent preventive therapy in pregnant women [12]. More surprisingly, this study found the use of CQ, which was last recommended in 2005 [10]. The study found that children whose parents had a primary education were more likely not using malaria chemoprophylaxis.

This study did not capture the information regarding the sources of information from parents who gave chemoprophylaxis to their children and how this practice is done. This highlights the need for Tanzania’s malaria treatment guidelines to specifically indicate if there is still a need to use chemoprophylaxis against malaria in SCD or not. Furthermore, an alternative drug should be recommended or screened in regions where malaria transmission is high to moderate, while advocating case management and use of insecticides in regions with low transmission [9, 13].

The study was conducted in the Dar es Salaam region where malaria prevalence is low [11]; this limits any conclusion since there are other regions with high malaria prevalence (10–20%). The conclusion from this study was based on recall by participants.

## Conclusion

This study found a small proportion of children with SCD were using malaria chemoprophylaxis. Of these, ACT was most used, followed by SP and CQ. This pattern of use indicates a lack of policy and recommendation as to which anti-malarial should be used for prevention of malaria in SCD [14] children. Since these children are vulnerable to malaria and they can travel to highly endemic regions in Tanzania, the Ministry of Health should specifically indicate treatment guidelines on the anti-malarials to use as prophylaxis in this group. This study was conducted in a low malaria transmission region; it is recommended that a similar study be conducted in areas with moderate to high malaria transmission.

## Data Availability

Raw data analysed in this study are available from the corresponding author on reasonable request.

## Abbreviations

ACT: artemisinin combination therapy
AOR: adjusted odds ratio
CQ: chloroquine
IQR: interquartile range
SCD: sickle cell disease
SP: sulfadoxine-pyrimethamine
SPSS: Statistical Package for Social Sciences
WHO: World Health Organization
